# Biomarkers and immunotherapy in endometrial cancer: mechanisms and clinical applications

**DOI:** 10.3389/fimmu.2025.1684549

**Published:** 2025-10-17

**Authors:** Lianfang Zhao, Xiaohong Li, Yaling Jing, Liping Tang, Fang Lin, Yongqiang Zhang, Yuqin Tang, Chuanliang Chen, Jiayan Yang, Xiaofang Liu, Jianhui Chen

**Affiliations:** ^1^ Department of Medical Genetics, Suining Central Hospital, Suining, Sichuan, China; ^2^ Institute of Cancer Research, Henan Academy of Innovations in Medical Science, Zhengzhou, Henan, China; ^3^ Department of Anesthesiology and Perioperative Medicine, Henan Provincial People’s Hospital, People’s Hospital of Zhengzhou University, Zhengzhou, China; ^4^ Clinical Bioinformatics Experimental Center, Henan Provincial People’s Hospital, Zhengzhou University, Zhengzhou, China

**Keywords:** endometrial cancer, immunotherapy, biomarkers, immune checkpoint inhibitors, combination therapy

## Abstract

Advanced endometrial cancer (EC) poses significant therapeutic challenges due to molecular heterogeneity and immune evasion. Immune checkpoint inhibitors (ICIs) show promise, particularly in mismatch repair-deficient (MMRd) and POLE-mutated subtypes, but resistance remains a barrier. This review synthesizes recent advances in biomarker-driven immunotherapy for EC, focusing on predictive biomarkers (e.g., LRP2, FANCE, MSH2, miRNA signatures), combination strategies (ICIs with anti-angiogenics or PARP inhibitors), and challenges in clinical translation. We highlight the impact of tumor microenvironment components, emerging technologies like machine learning, and future directions for personalized immunotherapy. Standardizing biomarker testing and optimizing trial designs will be critical to overcome resistance and improve outcomes.

## 1 Introduction

EC poses a growing public health burden, particularly in high-income countries where the incidence of EC has risen by 1-2% annually in high-income countries, while 5-year survival rates for advanced disease remain below 20% for advanced disease. While early-stage EC generally carries a favorable prognosis, aggressive subtypes (notably high-grade serous and carcinosarcoma) frequently exhibit treatment resistance and poor outcomes. This clinical heterogeneity, driven by molecular diversity and microenvironmental factors like obesity and hormonal influences, demands innovative management approaches (1).

The advent of immunotherapy has revolutionized EC treatment, particularly for MMRd and POLE-mutated subtypes. ICIs, particularly PD-1/PD-L1 inhibitors, have shown 40-60% response rates in MMRd and POLE-mutated EC subtypes, outperforming conventional therapies. However, intrinsic and acquired resistance mechanisms—often mediated by immunosuppressive tumor microenvironment (TME)—limit broader efficacy, underscoring the need for predictive biomarkers (2).

Molecular classification via The Proactive Molecular Risk Classifier for EC (ProMisE) classification identifies four molecular subtypes: mismatch repair-deficient (MMRd), POLE-mutated (POLEmut), p53 abnormal (p53abn), and no specific molecular profile (NSMP) exhibit differential immunotherapy responses. This stratification enables precision immunotherapy approaches while highlighting knowledge gaps in resistant subtypes (3).

Current research focuses on overcoming resistance through: Biomarker discovery (LRP2, FANCE mutations). Combination strategies (ICIs with PARP inhibitors/antiangiogenics). And microenvironment modulation (targeting Tregs/MDSCs). Ongoing clinical trials are evaluating these approaches, with preliminary data suggesting improved progression-free survival in advanced/recurrent disease (4–6). Overall, we summarize the diagnostic tools and supporting clinical evidence of the key biomarkers as a comprehensive table (**Table 1**).

**Table 1:** The key biomarkers information.

Biomarker	Diagnostic tools	Clinical evidence
POLE	NGS	High tumor mutation burden, favorable prognosis, significant lymphocyte infiltration, high likelihood of durable responses to checkpoint inhibitors (151).
PD-L1	IHC	Tumor microenvironment often exploits PD-L1 to evade immune surveillance (6, 28, 108, 109).
PD-1	IHC	Demonstrated efficacy in MSI-H/dMMR ECs, with high TMB increasing susceptibility to PD-1 blockade; enhances cancer cell recognition and elimination; durable responses including complete remissions observed (26–28).
miR-21	NGS	Elevated levels of miR-21 in serum have been associated with poor prognosis and advanced disease stages (107, 116, 152).
HE4	IHC	HE4 has shown promise in differentiating between benign and malignant endometrial lesions, with higher levels correlating with more aggressive disease (153).
CA-125	IHC	CA-125 has shown promise in differentiating between benign and malignant endometrial lesions, with higher levels correlating with more aggressive disease (154).
PTEN	IHC, NGS	Genetic mutations, particularly in the PTEN and TP53 genes, are common in endometrial tumors and can serve as indicators of tumor behavior (155).
TP53	NGS, IHC	High-grade tumors with TP53 mutations correlate with aggressive behavior and immunosuppressive microenvironment remodeling (6, 155).
HER2	IHC	Ongoing trial (NCT04551898) evaluating HER2-targeted CAR T-cells (156).
CCL20-CCR6	NGS	Mediates MDSC recruitment, representing a potential target for therapy (117, 122, 123).
ER/PR	IHC	Provides crucial prognostic and therapeutic insights; fundamental for biomarker detection (157).
KRAS	NGS	Used to inform immunotherapy response; ongoing trials for combination regimens to optimize treatment (32, 145).
CTLA-4	IHC	Multi-target therapeutic strategies for improved patient outcomes. Under investigation for use in combination regimens; promotes T-cell proliferation, offering a synergistic approach with PD-1 inhibitors (22, 23).
LAG-3	IHC	LAG-3 is a potential immunotherapeutic target of endometrial cancer. Clinical trials investigating the role of anti-LAG-3 antibodies, alone or in combination with other immunotherapies, are warranted (24).
TIM-3	IHC	A potential role for checkpoint inhibitors targeting TIM-3 in a subset of endometrial cancers (25).
CD39/CD73	IHC	Potential therapeutic target for restoring antitumor immunity (117, 122, 123).
SLC38A3	NGS	Correlates with prognosis and immunotherapy efficacy, critical for T-cell metabolism (96).
HLA genes	NGS	Hypermethylation of MHC genes impairs T-cell recognition, fostering an immunosuppressive environment (158).
Tregs (FoxP3+ Tregs)	IHC, Flow Cytometry	p53abn tumors cultivate immunosuppressive microenvironments, paving the way for combination therapies targeting checkpoint molecules and suppressive immune populations (38).
M2 Macrophages (CD163^+^ TAMs)	IHC, Flow Cytometry	M2 macrophages secrete IL-10 and TGF-β, inhibiting effector T cells and NK cells, promoting tumor progression. Elevated PD-L1 levels predict adverse outcomes in EC (52, 159).
FANCE	NGS, IHC	High FANCE tumors frequently demonstrate resistance to ICIs, ongoing research on its role in the tumor microenvironment and treatment resistance (86, 87).
Epigenetic modulators (EZH2, HDACs)	NGS	DNMT inhibitors are being explored to restore immune recognition in resistant tumors (132).
TILs	Flow cytometry	Enhanced treatment outcomes through the combination of immunotherapy and chemotherapy (106).
LRP2	IHC	Strong predictive value is part of LRP2 mutant signature (LMS), significantly improved survival in LMS-positive tumors post-immunotherapy, correlate with improved overall survival in immunotherapy patients (78, 160).
PMS2, MLH1, MSH2, MSH6,	IHC	Individuals carrying mutations in MutL homolog 1 (MLH1), MutS homolog 2 (MSH2), MutS homolog 6 (MSH6), or postmeiotic segregation increased 2 (PMS2) genes face an increased susceptibility to both endometrial and colorectal malignancies, with a lifetime risk ranging from 40% to 60% (69).

## 2 Mechanisms and clinical application of immunotherapy in endometrial cancer

### 2.1 Immunotherapy efficacy in EC varies based on molecular subtype

#### 2.1.1 Molecular classification and immunotherapy response

EC is a heterogeneous disease with distinct molecular subtypes that have significant implications for prognosis and treatment strategies. The ProMisE classification identifies four molecular subtypes: MMRd, POLEmut, p53abn and NSMP. Each subtype exhibits unique biological characteristics, mutation profiles, and clinical outcomes, which are crucial for tailoring therapeutic approaches. Recent studies have demonstrated that the MMRd subtype is often associated with high levels of tumor-infiltrating lymphocytes (TILs), indicating a robust immune response. This subtype is particularly responsive to ICIs, such as pembrolizumab and dostarlimab, which have been approved for treatment in patients with MMRd EC. The POLEmut subtype, characterized by a high tumor mutation burden and favorable prognosis, also shows promise for immunotherapy, as it tends to exhibit significant lymphocyte infiltration and a high likelihood of durable responses to checkpoint inhibitors (7–9). In contrast, the p53abn subtype is associated with a poor prognosis and is characterized by a high copy number alteration, which correlates with aggressive tumor behavior and resistance to standard therapies. The NSMP subtype, while having an intermediate prognosis, presents challenges in treatment due to its variable response to therapies (10, 11). The integration of molecular classification into clinical practice has highlighted the importance of personalized medicine in managing EC. For instance, the use of next-generation sequencing (NGS) for molecular classification has shown high concordance with traditional methods and can provide valuable prognostic information (12, 13). Moreover, studies suggest that molecular profiling could guide decisions regarding adjuvant therapies, allowing for de-escalation in low-risk subtypes such as POLEmut and MMRd, while intensifying treatment for high-risk subtypes like p53abn (14, 15). As research continues to evolve, understanding the molecular underpinnings of EC will be paramount in developing innovative therapeutic strategies and improving patient outcomes. Future clinical trials should focus on the distinct characteristics of each molecular subtype to optimize treatment regimens and enhance precision medicine approaches in EC management.

#### 2.1.2 Association of molecular subtypes with immune checkpoint molecule expression

Recent investigations have uncovered unique immune checkpoint expression profiles across four molecular subtypes of EC, these findings provide crucial insights for the advancement of immunotherapy. The PD-1/PD-L1 pathway displays subtype-specific expression patterns that correlate with the TME and therapeutic responses. MMRd and POLEmut subtypes are characterized by elevated programmed death-ligand 1 (PD-L1) expression and significant T cell infiltration, suggesting a favorable environment for checkpoint inhibition. Conversely, the p53abn tumors demonstrate unexpectedly high PD-L1 levels despite their immunosuppressive characteristics, indicating potential adaptive resistance mechanisms. NSMP tumors present moderate PD-L1 levels accompanied by heterogeneous immune infiltration (7, 8). The genomic instability inherent in the p53abn subtype is responsible for the upregulation of immune checkpoints, driven by neoantigen-associated inflammation (16). These aggressive tumors cultivate immunosuppressive microenvironments enriched in regulatory T cells (Tregs) and M2 macrophages, thereby creating avenues for combination therapies that target both checkpoint molecules and suppressive immune populations (17, 18). Notably, the co-expression of PD-L1 and Tregs in p53abn tumors establishes a self-perpetuating immunosuppressive circuit (19). The overexpression of PD-1 is indicative of exhausted T cells, a prevalent characteristic in EC attributed to persistent antigen exposure (20). Molecular subtyping indicates that PD-L1-positive high-grade serous carcinomas exhibit a superior response to checkpoint blockade compared to their low-grade counterparts (21). The co-expression of PD-L1 with CTLA-4, LAG-3, and TIM-3 suggests potential multi-target therapeutic strategies (22–25). The integration of precision medicine, utilizing molecular profiling alongside immune checkpoint evaluation, facilitates optimal patient stratification. Future research endeavors should aim to elucidate these mechanisms further and devise effective combination therapies directed at both tumor cells and their microenvironment (16). This molecular framework not only clarifies the heterogeneity of EC but also directs precision immunotherapy, with MMRd and POLEmut tumors emerging as prime candidates for ICIs.

### 2.2 Application of ICIs in EC

#### 2.2.1 Main ICIs drugs and their mechanisms

**Figure 1:**
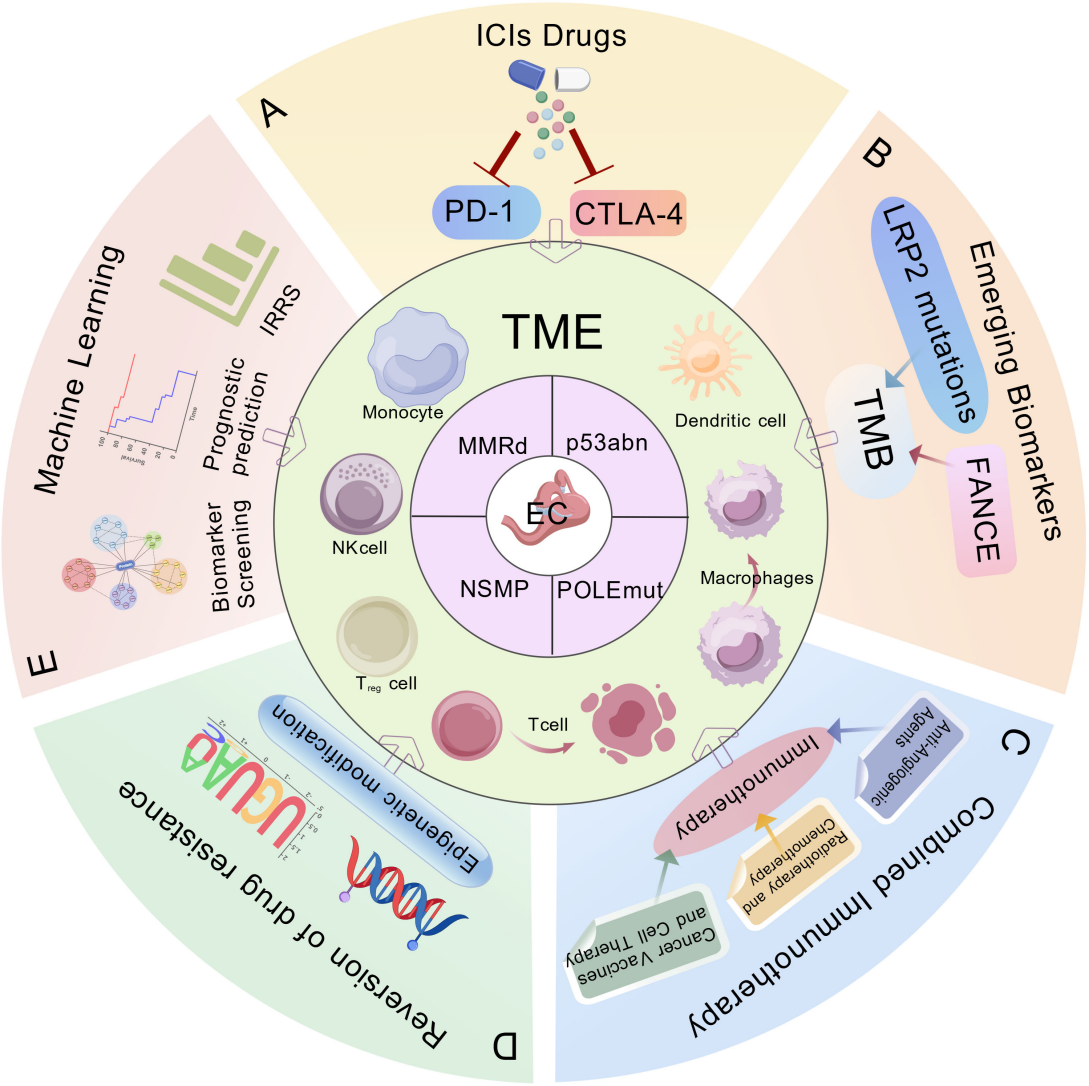
Biomarkers and immunotherapeutic strategies in endometrial cancer (EC).​​ The ProMisE molecular subtypes of endometrial cancer: mismatch repair-deficient (MMRd), POLE-mutated (POLEmut), p53 abnormal (p53abn), no specific molecular profile (NSMP). This schematic integrates key clinical applications including: **(A)** Immune checkpoint inhibitors (ICIs), **(B)** Emerging predictive biomarkers, **(C)** Combinatorial immunotherapy approaches, **(D)** Strategies to overcome therapeutic resistance, **(E)** Machine learning-assisted immune scoring systems for prognostic stratification. ProMisE, Proactive Molecular Risk Classifier for Endometrial Cancer; ICIs, Immune checkpoint inhibitors; PD-1, programmed death 1; CTLA-4, cytotoxic T lymphocyte associated antigen-4; TME, tumor microenvironment; FANCE, Fanconi anemia complementation group E; LRP2, low density lipoprotein-related protein 2; TMB, tumor mutational burden; IRRS, Immune Response-Related Scores.

ICIs, particularly PD-1 inhibitors such as pembrolizumab and nivolumab, have revolutionized EC treatment (**Figure 1A**). Pembrolizumab and nivolumab block PD-1, preventing its interaction with PD-L1/PD-L2 on tumor cells, thereby restoring T-cell-mediated antitumor activity. By disrupting this immunosuppressive axis, ICIs restore T-cell-mediated antitumor activity, enhancing cancer cell recognition and elimination (26). High tumor mutational burden (TMB) increases susceptibility to PD-1 blockade (27). The TME in EC often exploits PD-L1 expression to evade immune surveillance. Pembrolizumab and nivolumab counteract this by reinvigorating exhausted T-cells, leading to durable responses—including complete remissions in some cases evasion (28). Current research explores combination strategies (e.g. ICIs with chemotherapy or targeted therapies) to improve outcomes in advanced/recurrent disease (2, 29). Beyond PD-1 inhibitors, CTLA-4 inhibitors (e.g. ipilimumab) are being investigated in combination regimens. While PD-1 blockade enhances T-cell activation, CTLA-4 inhibition promotes T-cell proliferation, offering a synergistic approach to overcome resistance (22). Understanding these mechanisms is critical for optimizing treatment. ICIs represent a paradigm shift in EC therapy, with ongoing research poised to further refine their application and expand therapeutic potential.

#### 2.2.2 Clinical trial progress and efficacy assessment

Recent clinical trials have significantly advanced EC treatment through immunotherapy and combination strategies. Key phase II/III trials (RUBY [NCT03981796], NRG-GY018 [NCT03914612]) demonstrate improved overall survival when combining ICIs (pembrolizumab, dostarlimab) with chemotherapy in advanced/recurrent disease (2, 30). The RUBY trial (NCT03981796) demonstrated a 12-month PFS improvement with dostarlimab plus chemotherapy versus chemotherapy alone. These regimens enhance both response rates and survival outcomes compared to chemotherapy monotherapy. Biomarker-driven approaches using MSI status and TMB have become crucial for patient stratification. Recent studies showed pembrolizumab with lenvatinib is effective in MSI-H (microsatellite instability-high) advanced EC and conditionally approved for PD-L1-positive cervical cancer, with additive effects in MSS (microsatellite-stable) tumors, suggesting improved survival rates. Despite lower efficacy in MSS patients, combining therapies may enhance outcomes, necessitating further trials to optimize treatment and explore biomarkers (31–33). Efficacy assessment now incorporates immune-related biomarkers (PD-L1 expression, TILs) alongside traditional PFS/OS endpoints, improving patient selection (34, 35). Current research focuses on optimizing combination therapies through: Enhanced biomarker identification, and novel immunotherapy combinations, and improved understanding of tumor microenvironment interactions. This paradigm shift toward precision medicine in EC treatment is further complemented by the exploration of other ICIs, such as atezolizumab and avelumab (36, 37), which are also under evaluation for their potential benefits in this therapeutic landscape.

Recent studies evaluated pembrolizumab with lenvatinib in MSI-H and MSS tumors, showing significant efficacy in MSI-H advanced EC and conditional approval in PD-L1-positive cervical cancer. For MSS tumors, this combination therapy has shown additive effects, particularly in advanced EC, suggesting better survival rates compared to monotherapy in MSI-H tumors. Despite lower efficacy of ICIs in MSS patients, combining targeted therapy may improve outcomes. Further clinical trials are needed for MSS tumors to optimize treatment and confirm long-term effects, with future research focusing on biomarkers and effective combination therapies.

#### 2.2.3 Immune-related adverse reactions and management

Immune-related adverse events (irAEs) represent a critical challenge in immunotherapy for EC, occurring across multiple organ systems with varying severity. Common manifestations include dermatologic (rash, pruritus), gastrointestinal (colitis), endocrine (thyroid dysfunction), and pulmonary (pneumonitis) toxicities, resulting from unintended immune activation against healthy tissues (38). A recent systematic review and meta-analysis highlighted the varying rates of irAEs across different treatment regimens, suggesting that the combination of ICIs with other therapeutic modalities, such as chemotherapy or targeted therapies, may elevate the incidence of these adverse reactions. For instance, a study involving neoadjuvant therapy for non-small cell lung cancer reported that patients receiving combination therapy experienced a significantly higher frequency of treatment-related adverse events compared to those receiving ICIs alone, with grade 3 or higher irAEs occurring in approximately 25.7% of patients undergoing combined therapies (39, 40). Furthermore, specific combinations, such as anti-PD-1 agents with chemotherapy, have been shown to enhance both efficacy and the occurrence of irAEs. The data indicate that while these combinations can improve overall survival rates, they also necessitate close monitoring for adverse effects, particularly those that may arise early in the treatment course (41, 42). The observed incidence rates of irAEs can differ substantially based on the specific ICI employed; for example, anti-CTLA-4 therapies have been associated with higher rates of gastrointestinal events, whereas anti-PD-1/PD-L1 therapies frequently lead to pneumonitis and hepatotoxicity (43).

A retrospective analysis also revealed that the addition of targeted therapies, such as regorafenib, to ICIs could potentially enhance therapeutic outcomes without a corresponding increase in the severity of irAEs, indicating that careful selection of combination regimens may mitigate adverse effects while maximizing clinical benefits (40, 44). Management requires a tiered approach: Grade 1-2: Symptomatic treatment with continued ICI therapy under close monitoring. Grade 3-4: High-dose corticosteroids (prednisone 1–2 mg/kg/day) with potential addition of secondary immunosuppressants (infliximab for colitis, mycophenolate for hepatitis) (45). Life-threatening reactions: Permanent ICI discontinuation (46). Risk factors include a specific ICI regimen, with CTLA-4 inhibitors showing higher toxicity; pre-existing autoimmune conditions; and delayed onset of adverse events occurring weeks to months post-treatment (38). Optimal care involves: Baseline risk assessment, early recognition through vigilant monitoring, prompt intervention with appropriate immunosuppression, and multidisciplinary collaboration. Future research should focus on predictive biomarkers and targeted prevention strategies to maximize immunotherapy benefits while minimizing toxicity (38).

Overall, understanding the incidence rates of irAEs in the context of various ICI combinations is essential for optimizing patient management strategies and improving treatment tolerability. Continued research in this area is crucial for refining therapeutic protocols and enhancing patient safety in immunotherapy applications (39, 42).

### 2.3 The influence of the TME on the response to immunotherapy

#### 2.3.1 TILs and their prognostic

TILs, particularly CD8^+^ T cells, are key mediators of anti-tumor immunity and serve as important prognostic markers in EC. High CD8^+^ TIL density correlates with improved survival (HR 0.65; 95% CI 0.5-0.8) and predicts better response to PD-1 inhibitors. CD8^+^ T cells exert direct cytotoxic effects on tumor cells, and their higher density in the TME correlates with improved patient survival (47). Specific subsets, such as central and effector memory T cells, further enhance prognostic accuracy by associating with robust immune responses and better clinical outcomes (48). The infiltration of CD8^+^ T cells reflects both the immune competence of the host and the immunogenic properties of the tumor, which are modulated by mutational burden and immune checkpoint molecules such as PD-L1 (34). Furthermore, TIL levels are indicative of immunotherapy effectiveness, as EC patients with a high presence of CD8^+^ T cells demonstrate more favorable responses to PD-1/PD-L1 inhibitors like pembrolizumab (35). Conversely, low TIL infiltration is associated with worse prognoses and diminished treatment benefits (32). These observations highlight the clinical significance of immune profiling. However, it is essential to note that many studies investigating TILs were conducted on tumor types other than EC, which may lead to potential misinterpretations. In conclusion, CD8^+^ T cells are fundamental to anti-tumor immunity in EC, with their density and functional status offering critical prognostic insights. Future research should delve into the dynamics between the TME and TILs, aiming to optimize TIL-based strategies for personalized immunotherapy.

#### 2.3.2 Immunosuppressive cells and factors

The EC microenvironment harbors key immunosuppressive elements, including M2 macrophages and Tregs, which promote tumor progression. M2 macrophages secrete IL-10 and TGF-β, inhibiting effector T cells and NK cells, while tumor-derived signals drive their polarization (38, 49). Tregs maintain immune tolerance but impair anti-tumor responses by suppressing CD8^+^ T cell function, correlating with poor prognosis (50, 51). Targeting M2 macrophages (via CSF1R inhibitors) or Tregs (via anti-CTLA-4) may reverse immunosuppression. Immune checkpoint molecules further reinforce immunosuppression. PD-L1 expression on tumor cells induces T cell exhaustion upon PD-1 binding, and CTLA-4 enhances Treg activity, collectively facilitating immune evasion. In EC, elevated PD-L1 levels predict adverse outcomes (38, 52). Myeloid-derived suppressor cells (MDSCs) exacerbate this suppression through metabolic interference, releasing T cell-inhibitory metabolites (53, 54). These interconnected mechanisms create a permissive niche for tumor growth, highlighting the need for therapies targeting immunosuppressive pathways to restore anti-tumor immunity. The tumor-associated stroma plays a crucial role in influencing the response to immunotherapy in EC, as it comprises a complex network of various cell types, including fibroblasts, immune cells, and extracellular matrix components, which together create a unique microenvironment that can either promote or inhibit the effectiveness of therapeutic interventions. This stroma can modulate immune responses by secreting cytokines and growth factors that either enhance the recruitment and activation of immune cells or contribute to an immunosuppressive environment, thereby impacting the overall efficacy of immunotherapeutic strategies aimed at harnessing the body’s immune system to target and eliminate cancer cells.

#### 2.3.3 Metabolic reprogramming and immune regulation

Metabolic reprogramming significantly influences immune function and tumor progression in EC. Tumor cells exhibit enhanced glycolysis, depleting glucose and creating an immunosuppressive microenvironment that impairs TIL function (55, 56). This metabolic competition between tumor and immune cells exacerbates immune evasion. T cell activation requires glycolytic upregulation, but immunosuppressive metabolites (e.g., lactate, adenosine) in the TME inhibit effector T cells while promoting Treg differentiation (56, 57). Similarly, altered lipid metabolism in tumor-associated macrophages (TAMs) contributes to their pro-tumorigenic phenotype and T cell suppression (58, 59). Metabolic biomarkers like neutrophil-to-lymphocyte ratio (NLR) show prognostic value in EC immunotherapy response (60). Targeting these metabolic pathways, particularly when combined with immune checkpoint blockade, represents a promising therapeutic strategy (30, 61). Future research should focus on clinical translation of metabolic profiling to optimize personalized immunotherapy in EC.

### 2.4 Application of emerging biomarkers in immunotherapy

#### 2.4.1 TMB and its detection methods

TMB, quantifying somatic mutations per megabase, predicts immunotherapy response by increasing neoantigen formation. In EC, high TMB correlates with improved outcomes following immune checkpoint inhibition (62, 63). While NGS-based TMB assessment requires standardization, with thresholds of ≥10 mut/Mb typically defining high-TMB tumors (31, 62). Emerging evidence suggests microRNAs (miRNAs) may serve as surrogate TMB markers. Specific miRNA signatures correlate with TMB levels across cancers, potentially enabling liquid biopsy-based estimation (64). These miRNAs may modulate DNA repair pathways, influencing tumor mutation rates. Integrating miRNA profiling with conventional TMB analysis could refine immunotherapy selection, though clinical validation in larger cohorts is required (62, 64).

#### 2.4.2 Microsatellite instability and mismatch repair

The MSI-H status, stemming from the MMRd mechanism, is a crucial determinant in predicting responses to immunotherapy in EC. Tumors characterized by MSI-H are marked by a heightened mutational load and the generation of neoantigens, which significantly increases their susceptibility to ICIs such as pembrolizumab (65, 66). Approximately 30% of EC cases exhibit MSI-H characteristics, which may also suggest the presence of Lynch syndrome, thereby necessitating genetic counseling for affected individuals (67, 68). The evaluation of the expression of MMR proteins (MSH2, MSH6, MLH1, PMS2) through immunohistochemistry (IHC) serves as a reliable method for identifying dMMR tumors (69). These tumors often display unique clinical behaviors, such as elevated recurrence rates, yet paradoxically tend to respond more favorably to immunotherapy (65, 70). Among the core MMR proteins (MSH2, MSH6, MLH1, PMS2), MSH2 has been relatively well-studied in the context of genomic stability and tumorigenesis. Loss of MSH2 expression, similar to loss of other MMR proteins, can result in MSI, which is frequently observed in Lynch syndrome–associated tumors and also in a subset of endometrial cancers. MSI-H ECs, irrespective of the underlying defective MMR protein, generally exhibit high mutational burden and favorable responses to immune checkpoint blockade (71, 72). Interestingly, studies in colorectal and small bowel cancers have suggested that MSH2-deficient tumors may present with distinct clinicopathological traits (73–75). Although direct evidence in EC is limited, these observations imply that MSH2 alterations could hold comparable diagnostic and prognostic value in EC, and further investigation is warranted. Collectively, these findings highlight the importance of routine MMR protein evaluation in EC, with potential clinical utility in both risk stratification and immunotherapy guidance (76, 77). Overall, these assertions are supported by studies elucidating MSH2’s role in cancer pathology, emphasizing its clinical significance in diagnosis and treatment planning (66, 70). Hence, the implementation of routine MSI/MMR testing has become a standard practice in the management of EC, guiding therapeutic interventions and hereditary cancer risk evaluations. Future investigative efforts should prioritize refining combination strategies for dMMR tumors while simultaneously uncovering the underlying mechanisms that contribute to resistance against these therapies (65, 70).

#### 2.4.3 LRP2 mutations and their predictive value for immunotherapy

Emerging studies indicate that LRP2 mutations may not always be direct drivers of oncogenesis but could function as passenger mutations within the genomic landscape of highly mutated tumors—this perspective aligns with findings linking LRP2 mutations to increased TMB and T cell density, which influence the tumor microenvironment and response to immunotherapy (78, 79). Notably, this correlation is particularly relevant in EC: LRP2 mutations in EC are emerging as important biomarkers for immunotherapy response (**Figure 1B**), correlating with elevated TMB and MSI—both critical factors predictive of favorable immunotherapy outcomes (31, 78, 80).

Recent research further demonstrates that patients harboring LRP2 mutations (including those with EC) often exhibit higher TMB/MSI levels, and LRP2-mutated EC tumors show enhanced immune cell infiltration, frequently co-occurring with POLE and MSI-high subtypes (80, 81). To leverage this, the LRP2 mutant signature (LMS) combines LRP2 mutational status with immune gene expression patterns, exhibiting strong predictive value: patients with LMS-positive EC tumors have significantly improved survival following immunotherapy compared to LMS-negative cases, while high FANCE expression in these contexts predicts resistance and warrants combination strategies (64, 82).

Beyond predictive utility, LRP2 mutations may hold prognostic significance in EC—recent studies suggest they correlate with favorable outcomes in certain subtypes (e.g., MMR-deficient/MMRd and POLE-mutant/POLEmut EC), which are already recognized for distinct behaviors and treatment responses (78). Notably, Li et al. reported that in the EC cohort, patients harboring LRP2 mutations mostly belonged to the POLE and MSI-H subtypes and showed better prognosis. They further developed an LRP2 mutation signature (LMS), which was significantly associated with higher TMB, increased immune infiltration, and improved prognosis in patients receiving immunotherapy (78). Taken together, these findings underscore LRP2 alterations as a promising supplementary biomarker to refine patient stratification in EC, particularly within immunogenic subtypes such as POLEmut and MMRd, with future studies needed to directly compare their relative predictive power.

Understanding LRP2 mutations’ context—their role as both passenger events and modifiers of tumor behavior, alongside other genetic alterations and the tumor’s immune landscape—provides insights for personalized treatment. However, further validation is needed before routine clinical implementation: critical next steps include delineating whether LRP2 holds independent prognostic significance within MMRd/POLEmut EC subtypes and conducting comparative analyses to assess its prognostic capabilities against established biomarkers like MMRd and POLEmut status (78). Additionally, the potential benefit of LMS in EC—given the availability of MMR protein IHC testing—lies in refining patient stratification for treatment and enhancing the precision of immunotherapy deployment. Overall, these findings position LRP2 mutations as valuable tools for personalizing EC immunotherapy, emphasizing the need to consider both genetic and immunological dimensions of tumor behavior.

#### 2.4.4 The dual role of FANCE in DNA repair and immune evasion

FANCE, a key DNA repair gene in the Fanconi anemia pathway, exhibits dual functionality in EC. While essential for interstrand cross-link repair, aberrant FANCE expression promotes tumor progression and immune evasion through cell cycle dysregulation (83, 84). Elevated FANCE levels correlate with poor prognosis and immunotherapy resistance, potentially via modulation of immune checkpoint molecules (85). Clinically, FANCE expression shows promise as a predictive biomarker for immunotherapy response (**Figure 1B**). High FANCE tumors frequently demonstrate resistance to ICIs, suggesting its utility in guiding combination therapies (86, 87). Ongoing research focuses on elucidating how FANCE remodels the TME to mediate treatment resistance.

These findings position FANCE as both a therapeutic target and predictive tool, offering opportunities to develop strategies that overcome immunotherapy resistance in EC.

### 2.5 Immune scoring system and prognosis prediction assisted by machine learning

The Immune Response-Related Scores (IRRS) represent a significant advancement in EC prognosis and treatment stratification (**Figure 1E**). IRRS, derived from machine learning (ML) models, stratifies EC patients into high- and low-immunogenic groups, guiding immunotherapy selection (88, 89). Clinically, high IRRS correlates with improved survival, reflecting cytotoxic T cell infiltration and immune activation, as shown by its association with CD8^+^ TIL densities (90, 91). Conversely, low IRRS indicates TMEs and predicts poor immunotherapy response (32), establishing its dual predictive value. Moreover, IRRS elucidates resistance mechanisms by revealing interactions among immune checkpoints, regulatory genes, and tumor-intrinsic factors such as epigenetic modifications (89). This supports rational combination therapies by integrating IRRS with TMB or PD-L1 expression to optimize treatment selection (92, 93).

ML is pivotal in EC biomarker discovery, particularly for identifying immune-related genes that refine therapeutic strategies (**Figure 1E**). Multi-model fusion approaches integrate heterogeneous data sources, extracting biologically meaningful patterns from complex datasets. ML algorithms efficiently analyze high-dimensional genomic data to pinpoint biomarkers linked to patient outcomes and treatment responses, a crucial advancement given EC’s molecular heterogeneity. Ensemble methods (e.g., random forests, support vector machines) effectively stratify patients by genetic profiles, revealing biomarkers predictive of immunotherapy response (94, 95). Beyond classification, ML enables functional characterization of novel immune-related genes, such as SLC38A3, a solute carrier involved in amino acid transport critical for T-cell metabolism. ML-driven studies have elucidated the role of SLC38A3 in the TME, demonstrating its correlation with prognosis and immunotherapy efficacy (96). These findings highlight ML’s potential in uncovering biomarkers for personalized treatment. The synergy between ML and high-throughput technologies (e.g., RNA sequencing, proteomics) has further revolutionized biomarker discovery. ML-driven multi-omics analyses identify biomarkers reflecting tumor immune landscapes, offering insights into immune evasion and therapy resistance (97, 98). Such integrative approaches enhance understanding of tumor biology, advancing targeted therapies and precision oncology.

The amalgamation of immune scoring into clinical practice signifies a pivotal leap forward in individualized EC therapy. The TILs systems quantitatively evaluate the immune dynamics within the TME, delivering essential immunological insights to inform treatment decisions. Specifically, tumors characterized by a robust infiltration of (TIL) or heightened expression of PD-1/PD-L1 exhibit an augmented responsiveness to checkpoint inhibitors such as pembrolizumab, especially within dMMR subtypes (28, 32). In contrast, cases with lower immune scores may necessitate alternative approaches to overcome immune resistance, thereby facilitating more accurate therapeutic distribution while minimizing unnecessary treatment-related toxicity.

IRRS bridges the realms of computational biology and oncology by delineating the immune landscape of EC, with ongoing validation efforts propelling personalized immunotherapy forward. Machine learning-driven biomarker discovery is revolutionizing EC research, unveiling immune-related genes and furthering personalized medicine, ultimately enhancing diagnostic precision and patient outcomes.

Future advancements hinge on the creation of intelligent diagnostic platforms that leverage AI/ML to amalgamate immune scores with multi-omics data. Such integration holds the potential to unveil novel predictive signatures and refine the selection of immunotherapy (99, 100). Prospective trials that validate the predictive capacity of immune scoring will be essential in establishing clinical protocols, thereby charting a course for more precise, data-informed management of EC and improved patient outcomes. AI-enhanced liquid biopsy analysis allows for the real-time tracking of ctDNA fluctuations, fine-tuning adaptive immunotherapy strategies.

### 2.6 Clinical research progress of combined immunotherapy strategies

#### 2.6.1 Synergistic therapy: ICIs combined with anti-angiogenic agents

The integration of ICIs with anti-angiogenic agents signifies a noteworthy progression in the treatment of EC (**Figure 1C**). The combination of cabozantinib, a multi-targeted tyrosine kinase inhibitor, and nivolumab, a PD-1 inhibitor, exemplifies a synergistic effectiveness by concurrently impairing tumor blood supply through VEGF inhibition and rejuvenating T-cell activity via PD-1 blockade. This dual approach not only normalizes the TME but also amplifies immune-mediated tumor eradication, yielding superior clinical results compared to single-agent therapies (101). The immunomodulatory effects of this combination are intricate; anti-angiogenic medications modify the immunosuppressive tumor milieu by diminishing Tregs and MDSCs, thus enhancing the efficacy of ICIs and facilitating T-cell infiltration (102). Emerging biomarkers, which encompass immune cell signatures and cytokine profiles, may further streamline patient selection. Additionally, the amalgamation of established predictors like TMB and MSI status with angiogenic markers could refine treatment stratification (103). Additionally, integrating established predictors (e.g., TMB, MSI status) with angiogenic markers could optimize treatment stratification (104). The combination of lenvatinib and pembrolizumab achieved a 38% overall response rate (ORR) in microsatellite stable (MSS) EC, as demonstrated in the KEYNOTE-775 trial (105), effectively addressing traditional resistance to ICIs. While the exploration of ICIs in conjunction with cabozantinib has predominantly been conducted in other malignancies, including renal cell carcinoma and ovarian cancer, there remains a notable deficiency in data concerning its application in EC. Therefore, emphasis should be placed on the trial involving pembrolizumab and lenvatinib (KEYNOTE-775), which revealed a promising 38% ORR in MSS EC (105), suggesting a significant advancement in overcoming conventional ICI resistance. Although this combination was also assessed in a first-line context, the outcomes were not favorable. In conclusion, the synergy between ICIs and anti-angiogenic agents, as illustrated by the pembrolizumab-lenvatinib pairing, underscores the urgent need for further exploration within EC treatment protocols to improve therapeutic results.

#### 2.6.2 Synergistic effects of immunotherapy with radiotherapy and chemotherapy

The integration of immunotherapy with radiotherapy or chemotherapy significantly enhances the treatment of EC through multifaceted mechanisms (**Figure 1C**). The synergy between radiotherapy and immunotherapy is marked by the induction of immunogenic cell death, which liberates tumor antigens and activates dendritic cells, thereby priming systemic immunity. When administered sequentially, with immunotherapy following radiotherapy, tumor control is notably intensified, as evidenced by preclinical and clinical studies that indicate improved survival rates and diminished recurrence (5, 6). On the other hand, chemotherapy, despite its traditional role as an immunosuppressive agent, can actually augment ICIs by instigating immunogenic cell death, depleting immunosuppressive cells such as Tregs and MDSCs, and enhancing antigen presentation. For instance, the RUBY trial highlighted substantial progression-free survival advantages when dostarlimab was combined with chemotherapy in advanced cases (106, 107). Optimizing the timing of administration—whether concurrent or sequential—is crucial for achieving a balance between immune activation and the preservation of lymphocytes. Innovative strategies are currently being explored, such as predictive biomarkers (e.g., PD-L1, TILs), metronomic chemotherapy, and triple-combination regimens to maximize the synergistic effects (108, 109). This integrated approach capitalizes on immunogenic cell death and modulation of the TME to yield superior patient outcomes, with ongoing trials focused on refining sequencing, dosing regimens, and biomarker-guided personalization. When combined with ICIs, radiotherapy triggers immunogenic cell death, releasing tumor antigens that bolster systemic immune activation.

#### 2.6.3 Exploration of immunotherapy with cancer vaccines and cell therapy

Immunotherapy has revolutionized EC treatment, with cancer vaccines and adoptive cell therapy (ACT) emerging as particularly promising approaches (**Figure 1C**). Current vaccine platforms - including peptide-based, dendritic cell-based, and mRNA vaccines - aim to activate antitumor immunity but face challenges in neoantigen identification and delivery efficiency. Combination strategies with ICIs show potential to overcome these limitations, as demonstrated by a phase II trial (NCT03946358) where neoantigen vaccines combined with PD-1 blockade improved progression-free survival (110, 111). ACT approaches, including tumor-infiltrating lymphocyte therapy and CAR T-cells, offer direct tumor targeting capabilities. While successful in hematologic malignancies, their application in EC requires overcoming solid tumor microenvironment barriers. The limited efficacy of cancer vaccines in gynecologic malignancies has been a significant concern within the medical community. This issue is particularly evident from the findings of the OVAL and Vaccibody clinical trials (112, 113), which explored the potential of these innovative therapies in treating such challenging conditions. These trials have demonstrated that, although vaccines tailored for gynecologic cancers show encouraging preliminary results, the immune response elicited is insufficiently strong to achieve the desired therapeutic outcomes.

Current research focuses on enhancing T-cell engineering and combining ACT with chemotherapy or targeted agents to improve efficacy (114, 115). These modalities represent transformative pillars of EC immunotherapy. Ongoing trials (e.g., NCT04551898 evaluating HER2-targeted CAR T-cells) are advancing toward clinical implementation (1, 6). Future success will depend on personalized approaches tailored to individual tumor immunology, potentially redefining treatment for advanced or refractory disease.

### 2.7 Mechanisms of immunotherapy resistance and reversal strategies

#### 2.7.1 Tumor cell genetic and epigenetic changes

Genetic and epigenetic alterations in EC drive both tumorigenesis and immune evasion. High-grade tumors frequently exhibit TP53 mutations, which correlate with aggressive behavior and immunosuppressive microenvironment remodeling. These genetic changes upregulate immune checkpoint molecules like PD-L1, enabling immune escape (6). Epigenetic modifications, including DNA methylation and histone acetylation, further contribute to immune evasion by silencing tumor suppressor genes and antigen presentation machinery. Notably, hypermethylation of MHC genes impairs T-cell recognition, fostering an immunosuppressive niche (106). Emerging evidence highlights miRNAs as key regulators of these epigenetic changes, adding complexity to immune evasion mechanisms (107, 116). The interplay between genetic mutations and epigenetic modifications creates a multifaceted resistance landscape that challenges immunotherapy efficacy in EC (**Figure 1D**).

#### 2.7.2 Formation of TME

The TME is a key driver of EC progression and therapy resistance, shaped by infiltrating immunosuppressive cells—Tregs, MDSCs, and TAMs. Tregs suppress effector T cells via IL-10 and TGF-β, while MDSCs inhibit T cell activation through arginase activity and ROS production. TAMs often adopt an M2-like phenotype, promoting immune suppression and tissue remodeling. Together, these cells form a self-reinforcing immunosuppressive network that enables immune evasion and metastasis (117, 118). Metabolic reprogramming further amplifies immunosuppression. Tumor-derived adenosine and lactate impair T cell function, with adenosine receptor signaling directly suppressing cytotoxicity and lactate inducing acidification. Hypoxia-driven HIF activation exacerbates these effects by upregulating immunosuppressive pathways, creating a nutrient-deprived, immune-hostile niche (119–121). Key molecular mechanisms include the CD39/CD73-adenosine axis and CCL20-CCR6-mediated MDSC recruitment, both potential therapeutic targets for restoring antitumor immunity (117, 122, 123). In summary, the EC TME arises from synergistic cellular, metabolic, and signaling adaptations that fuel tumor progression and undermine immunotherapy. Combinatorial strategies targeting these interconnected mechanisms may improve patient outcomes.

#### 2.7.3 Novel therapeutic strategies to overcome treatment resistance in EC

Treatment resistance remains a major clinical challenge in EC. Recent advances in targeted therapies and rational combination regimens offer promising solutions. Key strategies include (1): Overcoming P-glycoprotein (P-gp)-mediated drug efflux through pharmacological inhibitors (e.g., verapamil) to restore chemosensitivity (124, 125) (2). Combining ICIs with cytotoxic agents for dual immune activation and direct tumor killing (126). (3) Employing multimodal approaches like anlotinib to reverse resistance by targeting epithelial-mesenchymal transition (EMT) and angiogenesis (127). (4) Combining ICIs with surgical treatment for oligoprogressive disease may be a promising method to improve prognosis (128). (5) Leveraging PARP inhibitor-ICI synergy in homologous recombination-deficient tumors (2), sulforaphane for obesity-associated cases (129). And targeting immunosuppressive pathways like CD73-mediated adenosine production. Future directions include precision combination therapies guided by molecular profiling, incorporating bispecific antibodies, oncolytic viruses, and epigenetic modulators (130, 131). Developing predictive biomarkers and optimizing treatment sequencing will be crucial for balancing efficacy and toxicity. Overcoming resistance requires integrated approaches combining targeted agents, immunotherapy, and TME modulation, underscoring the need for continued translational research and clinical trials. The relevance of the DUO-E trial should be highlighted when examining the combinations of ICIs with PARP inhibitors, particularly in the context of restoring immune recognition in resistant tumors through the use of epigenetic modulators such as DNA methyltransferase (DNMT) inhibitors (132).

### 2.8 Detection techniques and standardization of biomarkers for immunotherapy

#### 2.8.1 Advances in histological and molecular detection techniques

Recent technological advances have transformed EC diagnostics through improved histological and molecular detection methods. The combined application of immunohistochemistry (IHC), next-generation sequencing (NGS), and liquid biopsy has enhanced diagnostic accuracy, prognostic evaluation, and personalized treatment approaches. IHC remains fundamental for biomarker detection, with hormone receptor status (ER/PR) and p53 expression providing crucial prognostic and therapeutic insights. Multiplex IHC, enabling concurrent assessment of multiple biomarkers, further refines tumor classification and outcome prediction (133). NGS has revolutionized molecular characterization by detecting mutations (e.g., PTEN), copy number variations, and gene fusions, facilitating targeted therapy selection (52). Liquid biopsy, through ctDNA and exosome analysis, offers noninvasive tumor monitoring, early recurrence detection, and resistance mechanism identification (6). Multi-omics integration of genomic, transcriptomic, and proteomic data improves risk stratification and treatment response prediction, such as immunotherapy efficacy assessment when combined with IHC (134). Emerging approaches like dynamic network biomarker analysis evaluate molecular pathway interactions to predict therapeutic response and identify novel targets (106). These evolving technologies are redefining EC management by enhancing diagnostic precision and enabling tailored therapies, with ongoing advancements promising to further improve patient outcomes.

#### 2.8.2 Clinical application standards for biomarker testing

The clinical implementation of biomarker testing in EC encompasses four critical components: specimen collection timing, biological sample selection, test interpretation, and clinical integration. The most effective testing periods occur at three pivotal clinical phases: initial diagnosis, treatment strategizing, and assessment of therapeutic response. For example, evaluating the status of MSI and MMRd is crucial for establishing eligibility for immunotherapy in advanced cases. While tumor biopsies are considered the gold standard for genetic profiling, liquid biopsies offer significant advantages in terms of longitudinal monitoring capabilities (6). Accurate interpretation of biomarkers necessitates that clinicians assimilate biological mechanisms with clinical implications, correlating the findings with other diagnostic metrics to inform treatment choices. Distinct biomarker patterns may signal disease aggressiveness or forecast therapeutic response (106). Ensuring standardized testing protocols is vital for achieving reproducibility of results across various institutions, necessitating strict compliance with specimen processing, assay performance, and documentation standards. Clinical decision support systems further refine this approach by generating evidence-based, patient-specific recommendations (107). The routine incorporation of biomarker testing facilitates personalized treatment strategies and enhances patient outcomes. To fully harness these advantages in light of rapid advancements, continuous professional development remains critical (134).

#### 2.8.3 Challenges in standardization and quality control

While biomarker integration has advanced EC immunotherapy, standardization and quality control of biomarker testing remain significant challenges. Assay variability across different platforms can critically affect clinical decisions and treatment outcomes. A prominent example is PD-L1 expression assessment, where discrepancies in antibody clones, detection methods, and scoring systems lead to inconsistent results, complicating immune checkpoint inhibitor eligibility determinations (97). This highlights the pressing need for standardized testing protocols. Establishing international consensus guidelines is essential to unify biomarker testing methodologies, including specimen processing, assay validation, and result interpretation. Comprehensive quality control measures must encompass the entire testing process - from pre-analytical specimen handling to analytical procedures and post-analytical reporting. Implementing robust quality management systems can significantly improve inter-laboratory reproducibility (64). with practical solutions including standardized control samples and regular proficiency testing. Continuous education for laboratory personnel on evolving biomarker technologies and quality assurance protocols is equally crucial. Although biomarker-guided immunotherapy shows great promise for EC treatment, addressing standardization challenges through international guidelines and rigorous quality control remains imperative to ensure reliable testing and optimal patient outcomes.

### 2.9 Differences in immunotherapy application among EC subtypes

#### 2.9.1 Immune sensitivity of MMRd and POLEmut

MMRd and POLEmut EC exhibit remarkable immunogenicity and a pronounced responsiveness to immunotherapy. Mutations within the POLE exonuclease domain elicit a hypermutated phenotype characterized by an abundance of neoantigens, which facilitates vigorous T cell infiltration and fosters an immunologically “hot” tumor microenvironment. Likewise, MMRd tumors present a significant TMB attributed to impaired MMR mechanisms. Both subtypes demonstrate notably enhanced responses to PD-1/PD-L1 inhibitors in comparison to microsatellite-stable tumors, yielding superior overall survival rates in clinical studies (135, 136). These immunogenic subtypes are marked by elevated PD-L1 expression and distinct immune-related gene signatures, which include heightened markers of T cell activation. While POLE mutations promote ongoing neoantigen presentation and immune surveillance, MMRd tumors also sustain high immunogenicity. Thus, the molecular characteristics of both types serve as validated predictive biomarkers for immunotherapy efficacy (137, 138). Despite generally positive outcomes, there exists variability in response within these subtypes, indicating the presence of residual immune evasion mechanisms. Transcriptomic evaluations reveal discrepancies in immune cell composition and functional states within the TME that may affect therapeutic effectiveness (139, 140). Further investigation is crucial to refine immunotherapy strategies tailored for these distinct yet highly immunogenic EC subtypes. Patients with rare histologies (serous, clear cell, carcinosarcoma) were included in pivotal clinical trials (RUBY [NCT03981796], NRG-GY018 [NCT03914612]). Both trials included the rare aggressive histologies of serous and clear cell carcinoma. Carcinosarcoma was only included in the RUBY trial. While the primary results grouped them into the larger MMRp population, subsequent data strongly supports the use of immuno-chemo combination therapy in these specific rare histologies, fundamentally changing the standard of care for this patient population (2, 30).

#### 2.9.2 P53abn type and NSMP subtype immunotherapy challenges

The p53 abnormal and NSMP EC subtypes present unique immunotherapy challenges due to their intrinsic biological features and immune evasion mechanisms. p53 mutations impair tumor suppressor function, correlating with aggressive behavior and poor outcomes. These tumors often exhibit immune escape through MHC class I downregulation (impairing T cell recognition) and PD-L1 upregulation, creating an immunosuppressive microenvironment that limits immunotherapy efficacy (141, 142). The NSMP subtype poses additional challenges due to its molecular heterogeneity and lack of defining genetic alterations. Encompassing tumors not meeting other molecular classifications, it shows variable treatment responses. The absence of reliable biomarkers complicates immunotherapy selection, though emerging markers like L1CAM may aid patient stratification (143, 144). Current strategies focus on enhancing immunogenicity through combination therapies, such as ICIs with MHC-restoring agents or microenvironment-modulating drugs.

#### 2.9.3 High-risk non-endocrine and other rare subtypes

Immunotherapy shows promise for high-risk non-endocrine and rare EC subtypes (e.g., serous, clear cell, carcinosarcoma), which pose therapeutic challenges due to their aggressive biology and poor prognosis. ICIs are under active investigation, particularly anti-PD-1 agents like pembrolizumab in dMMR/MSI-H tumors—features variably present across subtypes. Combination strategies (ICIs with lenvatinib or chemotherapy) have demonstrated improved survival in trials such as RUBY and NRG-GY018 for advanced/recurrent disease. However, biological heterogeneity necessitates better predictive biomarkers for patient selection. Ongoing trials are evaluating novel ICI combinations with antiangiogenics and targeted therapies, alongside genomic profiling to elucidate response and resistance mechanisms (2, 30). Precision medicine approaches are urgently needed for these molecularly diverse tumors. Current strategies employ molecular characterization (genomic sequencing, biomarker assessment) to guide therapy, with KRAS mutations, TMB, and dMMR status informing immunotherapy response. Liquid biopsy (ctDNA analysis) enables dynamic treatment monitoring and residual disease detection. Research now focuses on optimizing combination regimens to overcome resistance and address heterogeneity, aiming to personalize treatment while preserving quality of life (32, 145).

### 2.10 Future outlook: innovative directions of precision immunotherapy

#### 2.10.1 Multidimensional biomarker integration and dynamic monitoring

Integrative analysis of genomic, immunomic, and metabolomic biomarkers is transforming EC management. This approach simultaneously evaluates genetic alterations, immune microenvironment features, and metabolic reprogramming to better understand tumor biology. Genomic profiling identifies actionable mutations for targeted therapy, while immunomic analysis reveals tumor-immune interactions. Metabolomic data uncover therapeutic vulnerabilities through metabolic pathway analysis. Such multidimensional assessment improves patient stratification and identifies novel predictive signatures for treatment response (107, 134). Dynamic monitoring has become essential in precision oncology. Serial liquid biopsies (particularly ctDNA analysis) enable sensitive detection of residual disease and early recurrence, allowing timely treatment adjustments. Advanced MRI and PET provide complementary spatial and functional response data, offering a comprehensive view of therapeutic efficacy (6, 106). Artificial intelligence enhances analysis of these complex datasets. ML algorithms detect subtle patterns across biomarker platforms, generating increasingly accurate predictive models. These adaptive systems continuously improve with new clinical data, particularly in predicting immunotherapy responses and optimizing combination strategies (146, 147). In summary, integrating multidimensional biomarkers with dynamic monitoring enables truly personalized treatment by accounting for tumor complexity. These evolving approaches promise to enhance diagnostic precision, therapeutic outcomes, and patient survival in EC.

#### 2.10.2 Discovery and validation of novel immune regulatory targets

Recent advances in EC immunotherapy focus on next-generation ICIs and immune-metabolic interactions. While PD-1/CTLA-4 inhibitors have revolutionized treatment, variable responses highlight the need for better immune regulation understanding. Current biomarkers (PD-L1, TMB) show inconsistent predictive value, necessitating additional markers for optimal patient selection. Immune metabolism research has uncovered new therapeutic opportunities. Metabolic reprogramming of T cells critically impacts immunotherapy outcomes, with glycolysis and oxidative phosphorylation pathways modulating T cell function. Preclinical studies show metabolic modulators can enhance T cell activity and counteract tumor immunosuppression. The combination of checkpoint blockade with metabolic modulation shows particular promise for advanced/resistant cases. Ongoing clinical trials aim to establish novel protocols for rapid clinical translation (64, 97, 148). These discoveries in both checkpoint molecules and metabolic pathways are expanding treatment options, enabling more precise immunotherapy approaches for EC.

#### 2.10.3 Artificial intelligence and big data-driven immunotherapy decision-making

AI and big data analytics are transforming EC immunotherapy through ML and multi-center data integration. Advanced algorithms analyze multi-omics data (genomic, proteomic, clinical) to predict immunotherapy responses and guide personalized treatment. These systems detect subtle patterns in patient data that correlate with tumor responsiveness - crucial given EC heterogeneity. AI tools enable precise patient stratification and continuously refine treatment protocols as new evidence emerges (97, 148). Multi-center clinical data repositories aggregate diverse patient information (demographics, treatment histories, outcomes), facilitating population-level analysis and robust clinical trials of novel immunotherapy combinations. These datasets support development of predictive models that guide therapy based on individual tumor biology (64, 149). AI-powered liquid biopsy analysis (ctDNA, CTCs) provides real-time monitoring of tumor evolution and treatment response. This approach enables early detection of residual disease and recurrence, significantly impacting clinical outcomes. Combining AI with liquid biopsy creates adaptive immunotherapy strategies responsive to tumor dynamics (64, 150). In summary, AI and big data synergistically enhance biomarker discovery, treatment personalization, and decision support in EC immunotherapy. These technologies are becoming central to optimizing precision oncology care, with ongoing research refining best practices (97, 148).

#### 2.10.4 Innovative clinical trial designs

Oncology clinical trial design is evolving with novel approaches to enhance drug development efficiency and outcomes. Key innovations include biomarker-driven precision enrollment, leveraging molecular characteristics like MSI status and PD-L1 expression in EC to optimize patient stratification. This focus on high-response subgroups improves trial success and resource utilization. Adaptive designs further refine protocols through interim analyses, accelerating effective therapy identification and enabling personalized strategies—particularly valuable in advanced EC where chemotherapy often fails.

Modern trials now emphasize comprehensive efficacy assessments beyond survival metrics, incorporating patient-reported outcomes and quality-of-life data to better evaluate treatment impact. Studies of recurrent disease (e.g., ICIs plus antiangiogenic agents) highlight response variability linked to tumor biology and immune microenvironment, informing targeted interventions.

Liquid biopsy technology advances trial methodology by noninvasively monitoring circulating tumor DNA (ctDNA), enabling real-time response assessment and resistance mechanism insights. In EC trials, serial ctDNA analysis guides therapeutic adjustments, enhancing flexibility and personalization. Future studies should focus on overcoming resistance in p53-abnormal and NSMP subtypes through combination therapies targeting both tumor cells and the immunosuppressive microenvironment.

These innovations—precision enrollment, adaptive protocols, and multidimensional assessments—are transforming oncology research. Their application in EC addresses traditional limitations, paving the way for more effective, patient-specific therapies. Continued evolution of these methodologies will be critical for improving outcomes in this complex malignancy.

## 3 Conclusion

Immunotherapy has redefined EC treatment, with molecular subtyping and biomarker integration enabling precision medicine. This progress reflects the integration of basic research, clinical translation, and personalized care, setting a new oncologic standard. Key biomarkers—LRP2, FANCE, MSH2, and miRNA signatures—now enhance immunotherapy response prediction and patient selection, optimizing efficacy and resource utilization.

Challenges remain, particularly the complexity of tumor microenvironment and immune evasion mechanisms limiting broader application. Overcoming heterogeneity of EC requires innovative immunotherapy combinations with complementary treatments, a promising direction to address resistance and improve outcomes.

ML and immune scoring systems have advanced prognostic accuracy and personalized planning. Big data analytics and computational modeling refine tumor behavior analysis, enabling precise stratification and clinical decisions that balance survival benefits with quality of life.

Future progress hinges on multi-omics integration, real-time response monitoring, and novel target discovery. Standardized biomarker testing and innovative trial designs are crucial for clinical translation. As tumor-immune insights deepen, these efforts will refine immunotherapeutic strategies.

In summary, while immunotherapy has redefined EC care, its full potential demands sustained interdisciplinary collaboration. Future efforts must focus on multi-omics integration, dynamic monitoring, and innovative trial designs to unlock the full potential of immunotherapy in EC.
